# Development and Validation of an Instrument to Assess Endodontists' Knowledge, Attitudes, and Practices Regarding Cone‐Beam Computed Tomography

**DOI:** 10.1111/aej.70075

**Published:** 2026-03-29

**Authors:** Lisa Morais Fernandes Oliveira, Francielle Silvestre Verner, Pedro Henrique Berbert de Carvalho, Rafael Binato Junqueira

**Affiliations:** ^1^ Postgraduate Program in Applied Health Sciences (PPGCAS) Federal University of Juiz de Fora (UFJF) Governador Valadares Minas Gerais Brazil; ^2^ Division of Oral Radiology, Department of Dentistry Federal University of Juiz de Fora (UFJF) Governador Valadares Minas Gerais Brazil; ^3^ NICTA, Body Image and Eating Disorders Research Group Federal University of Juiz de Fora Governador Valadares Minas Gerais Brazil; ^4^ Department of Dentistry, Division of Endodontics Federal University of Juiz de Fora (UFJF) Governador Valadares Minas Gerais Brazil

**Keywords:** attitudes, cone‐beam computed tomography, knowledge, practices, psychometry

## Abstract

There is a scarcity of data regarding knowledge, attitudes and practices (KAP) of endodontists about cone‐beam computed tomography (CBCT), which may be related to the lack of valid and reliable measures. The present study aimed to develop and validate a KAP measure about CBCT. The developed measure showed good content validity and was administered to 506 endodontists. Exploratory and confirmatory factor analysis revealed a factorial structure composed of three factors (KAP) with 13 items. The measure showed evidence of good internal consistency and discriminant validity, being able to accurately discriminate KAP according to the length of experience and level of education. Taken together, results provided evidence of good psychometric properties of a new KAP measure regarding CBCT, allowing future research in the field.

## 1 Introduction

Endodontic diagnosis is formulated based on the patient's main complaint, medical history, professional experience, risk and benefit assessments, costs, prognosis, and treatment possibilities [1, 2]. Radiographic examination plays an important role in this process, supporting the diagnosis in pre‐, intra‐, and postoperative evaluations [1, 2]. However, this exam presents limitations such as difficulties in interpreting planar images, geometric distortion, anatomical noise, and masking periapical lesions with less than 7.1% of cortical bone loss [1, 3, 4, 5]. When digital radiography does not provide sufficient information, cone‐beam computed tomography (CBCT) gives additional details through three‐dimensional reconstruction of the area of interest [5].

According to the joint recommendation on the use of CBCT in Endodontics of the American Association of Endodontists (AAE) and the American Academy of Oral and Maxillofacial Radiology (AAOMR) [1], CBCT should be prioritized when patients present with contradictory or nonspecific clinical signs and symptoms associated with untreated or previously endodontically treated teeth.

The use of CBCT by endodontists has been evaluated in several countries, obtaining adherence of 26%–80%, depending on the region [6]. There are few studies in literature measuring the level of knowledge, attitudes, and practices (KAP) of endodontists regarding the adoption of CBCT [7]. This can be attributed to the absence of adequate measuring instruments, i.e., psychometrically evaluated, that allow such an assessment to occur in a valid and reliable manner.

The development and application of constructs assessing characteristics that cannot be measured directly are common in health practice and research [8, 9]. To ensure reliability in the features assessed and in subsequent comparisons and inferences, the creation of instruments requires a rigorous development process, and psychometric tests are necessary to assess their validity and reliability, ensuring that “subjective” phenomena are captured properly [8, 9].

If the questionnaire validation process is not implemented, there is a risk of inaccurate or biased data, vague conclusions, and research with results of questionable value [8, 9]. The instrument that seeks to measure KAP fits the definition of characteristics that cannot be measured directly.

Previous studies have evaluated jointly or separately KAP in relation to CBCT, but did not perform psychometric analyses [7]. Others have cited the measurement of properties such as knowledge and attitudes, but without due essay [5, 6, 10, 11]. The application of questionnaires about CBCT was also reported but did not mention the use of any type of validity of the instruments [3, 4, 12, 13, 14, 15]. Furthermore, no associations were found between endodontists' KAP in relation to CBCT and their length of experience or level of education. Thus, there is an important gap in literature regarding studies that have used reliable and reproducible methods for creating and validating questionnaires about imaging exams in Endodontics.

Considering the importance of CBCT in the clinical practice of endodontists and the need to investigate the relationship of specialists with this exam, the aim in this study was to develop and evaluate the evidence of validity and reliability of an instrument for measuring knowledge, attitudes and practices of endodontists in relation to CBCT.

## 2 Materials and Methods

### 2.1 Study Design and Ethical Considerations

**FIGURE 1:**
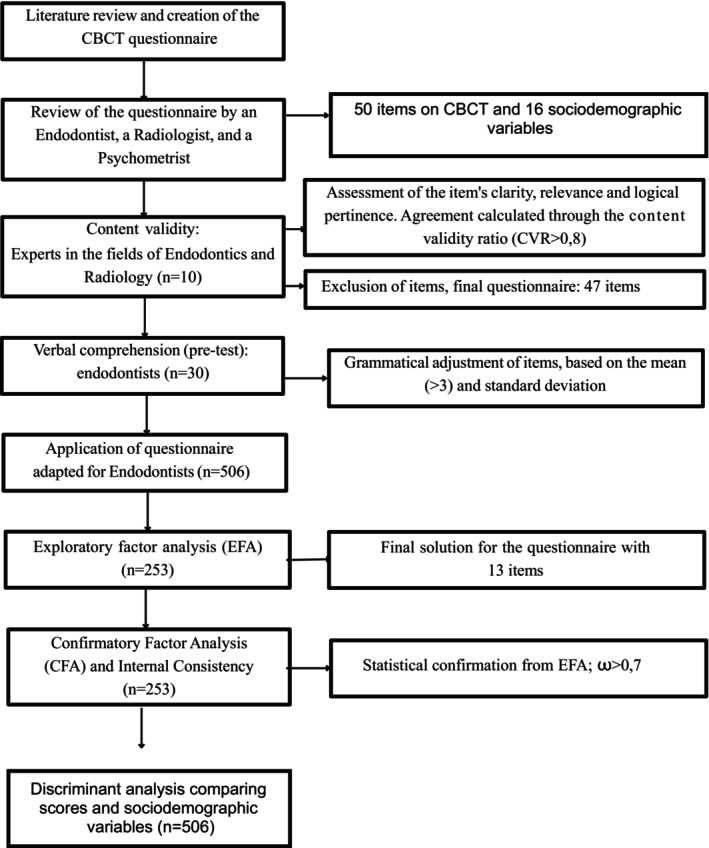
Flowchart summarizing the study.

This is a cross‐sectional quantitative methodological study, which followed the STROBE checklist (steps are summarized in Figure 1). The development process was divided into four phases: item generation, theoretical item analysis, empirical analysis, and psychometric analysis [16, 17]. To achieve the proposed objectives, one KAP (knowledge, attitudes, and practices) measurement instrument and a sociodemographic questionnaire were developed. This study was approved by the local Research Ethics Committee (protocol n. 7.289.945) in December 2024.

### 2.2 Inclusion Criteria

The inclusion criteria followed the stages of instrument construction:

– Theoretical analysis of items: experts, researchers and/or health professionals working in the field of Endodontics and/or Radiology and instrument validation.
– Empirical and psychometric analysis of items: Brazilian specialists in Endodontics.

### 2.3 Development of Questionnaires

The initial construction of the items was based on a literature review, considering studies on CBCT. The content was reviewed by an endodontist (R.B.J.), a radiologist (F.S.V.), and a psychometrist (P.H.B.C.), with at least 10 years of experience [16, 17]. Responses to the items were measured using a 5‐point *likert* scale, with the options “I strongly agree”, “I agree”, “I neither agree nor disagree”, “I disagree” and “I strongly disagree”.

For the present study, “knowledge” is understood as the theoretical understanding of professionals about imaging technologies (operating principles, indications, limitations); “attitudes” as issues related to beliefs, perceptions about the effectiveness and safety of technologies, besides the importance attributed to the use of advanced imaging techniques; and “practices” as effective actions of professionals in daily clinical practice (frequency of use, the integration of these tools in diagnostics and treatment planning) [18].

In the second stage, the instrument was submitted to the evaluation of ten experts, researchers and/or health professionals working in the field of Endodontics and/or Radiology and instrument validation. This evaluation included the analysis of item clarity, practical relevance, and logical pertinence [17, 19]. To calculate content validity, the Content Validity Ratio (CVR > 0.8) measured the percentage of agreement among experts on specific aspects of the instrument and items [20].

The items were then administered to a sample of the target population (pre‐test) for semantic analysis, consisting of 30 endodontists. Each participant assessed the adequacy and clarity of the language and their understanding of the items [19]. The verbal comprehension model used a 5‐point scale, and statistical analysis was performed by describing the mean (> 3) and standard deviation of each item [21].

### 2.4 Data Collection

After adjusting the instrument, it was applied to a new sample of endodontists from March to August 2025. The sample was calculated considering the proportion of five participants for each item created [22]. In addition to the specific instrument, a sociodemographic questionnaire with data on gender, age, time working as an Endodontist, region of Brazil in which the patient works, level of education, and questions about contact with CBCT was used to characterize the sample.

The instrument was administered via Google Forms (Alphabet Co., Mountain View, California, USA). Participants were invited to participate voluntarily through social media and messaging apps. Before receiving access to the questionnaires, participants agreed to participate through the Informed Consent Form (ICF).

### 2.5 Empirical and Psychometric Analysis

In the third and fourth phases (empirical and psychometric analysis), the individual performances of each item were verified to define which ones were maintained in the final version [18]. To assess the validity of the construct, exploratory factor analysis (EFA) and confirmatory factor analysis (CFA) were performed. Discriminant validity was also assessed by comparing the scores obtained by endodontists on the instrument in relation to the length of clinical practice and level of education. Finally, the reliability of the instrument was assessed through internal consistency analysis.

### 2.6 Exploratory Factor Analysis

In EFA, Bartlett's test of sphericity (*p* < 0.05) and Kaiser‐Meyer‐Olkin measure (KMO > 0.80) verified the adequacy of the data for factor analysis [23]. The extraction method used was principal axis factoring, oblique Oblimin factor rotation, and the polychoric correlation matrix. To define the factors, the Kaiser criterion (eigenvalue ≥ 1.0), scree‐plot and parallel analysis were performed. Item retention considered items with a factor loading (*λ*) greater than 0.40, and items with a factor loading greater than 0.32 in more than one factor were considered cross‐loading [22]. Total explained variance of the construct was considered relevant from 30% to 40% [22].

### 2.7 Confirmatory Factor Analysis

CFA was performed to confirm the factor structure obtained in the EFA. The robust diagonally weighted least squares method was used [23]. The adequacy of the model was assessed by the chi‐square test (*χ* ^2^) divided by the degrees of freedom (*χ* ^2^/df < 5) and by multiple fit indices: Comparative Fit Index (CFI~0.95), Tucker‐Lewis Index (TLI~0.95), Standardized Root Mean Square Residual (SRMR < 0.08) and the Root Mean Square Error Approximation (RMSEA < 0.08 [90% CI = 0.08–0.10]) [22]. The model fit by the model modification indices Langrange was inspected to verify cases with indices higher than 11 [23]. The factor loading of the items was evaluated and considered adequate if greater than 0.40.

To analyze internal consistency, McDonald's Omega coefficient (*ω*) (> 0.70) was calculated for each of the factors that comprised the instrument [19].

### 2.8 Discriminant Analysis

Discriminant analysis was used to compare the scores obtained by endodontists on the instrument in relation to length of clinical practice and level of education. Regarding length of practice, the analysis was performed using the Mann–Whitney *U* test (considering the non‐normal distribution of the data, *p* < 0.05); the normality of the data was tested by the Shapiro–Wilk test and the homogeneity of variance by the Levene's test. For the Mann–Whitney *U* test, the Cohen (1988) [24] effect size was calculated, considering the following values: small effect (≤ 0.10), medium (~0.30), and large (~0.50).

The Kruskal–Wallis test was used for comparisons involving educational level, and Dunn's post hoc test was used for pairwise comparisons. For the Kruskal–Wallis test, the reference for the effect size was epsilon squared (*ε* ^2^) = 0.01 – < 0.08 (small), 0.08 – < 0.26 (medium), ≥ 0.26 (large) [25].

All data were transposed into JASP software v. 0.19.1, with a significant level of 5% (*p* < 0.05).

## 3 Results

### 3.1 Content Validity

After ten experts evaluated the instrument for clarity, logical relevance, and item relevance, the responses were analysed. Two questions with CVR below the stipulated level were retained because the researchers considered them relevant. Based on the experts' considerations, four questions were rewritten to improve clarity, and twelve were excluded. In the determination of factors by the experts, 22 questions had an agreement rate above 80% and 25 had an agreement rate below the stipulated level (< 0.80). Of these, two were adjusted to improve the rate and the other 23 were maintained based on the clarity, logical relevance, and relevance indices, as the factors would be analysed by EFA.

### 3.2 Verbal Comprehension

The revised version of the instrument underwent semantic analysis to ensure comprehension among different professionals, involving a group of 30 endodontists. Then, 11 questions were adjusted to increase clarity for the target group. The adjustments consisted of adjusting or changing wording to improve item comprehension. The final version of the instrument consisted of 47 items categorized into three factors: Knowledge (17 items), Attitudes (18 items), and Practices (12 items).

### 3.3 Descriptive Analysis and Characterization of the Sample

A total of 506 endodontists participated, with no missing data. The sample was randomly divided in half (*n* = 253) for the EFA and CFA analyses. The mean age was 35.79 years (ranging from 22 to 68) and the mean length of experience as an endodontist was 9.39 years (ranging from 1 year or less to 42). Most endodontists received some instruction about CBCT only during their specialization, did not have a CBCT scanner in their workplace, and had been using it for between 1 and 9 years. Further details are presented in Table 1.

**TABLE 1:** Descriptive data of the sample that responded to the cone beam computed tomography (CBCT) questionnaire (*n* = 506).

Variables	Mean (SD)
Age (years)	35.79 (10.12)
Length of experience (years)	9.39 (9.13)
	***N* (%)**
Sex	
Male	160 (31.62)
Female	346 (68.38)
High level of education	
Specialization	318 (62.85)
Master's degree	110 (21.74)
Doctorate	71 (14.03)
Postdoctoral	7 (1.38)
Work sector (most of the time)	
Public	62 (12.25)
Private	444 (87.75)
Region of Brazil	
Southeast	232 (45.85)
South	111 (21.94)
Northeast	97 (19.17)
Central‐West	45 (8.89)
North	21 (4.15)
**CBCT**	
Did you have any classes or guidance on CBCT during your undergraduate degree?	
Yes	217 (42.89)
I don't remember	62 (12.25)
No	227 (44.86)
During your specialization, did you receive any instruction on CBCT?	
Yes	375 (74.11)
I don't remember	29 (5.73)
No	102 (20.16)
Have you ever taken a specific course on CBCT in Endodontics?	
Yes	152 (30.04)
I don't remember	11 (2.17)
No	343 (67.79)
I have the CBCT scanner in my office.	
Yes	36 (7.12)
No	460 (90.91)
I do not use CBCT in my clinical routine	10 (1.98)
How long have you been using CBCT? (in years)	
< 1	64 (12.65)
Between 1 and 9	352 (69.57)
Between 10 and 19	61 (12.06)
> 20	4 (0.79)
I do not use CBCT	25 (4.94)

### 3.4 Exploratory Factor Analysis

The KMO index = 0.78 and Bartlett's Sphericity Test (*χ* ^2^ (78) = 1197.877; *p* < 0.001) indicated the possibility of factoring the KAP instrument. The first factor solution identified consisted of nine factors: one factor with items that presented *λ* < 0.40, two factors containing only one item, and four factors with two items each. Given the problems presented by the model, the number of factors was manually reduced and analysed seeking the best fit, being respectively reduced to 8, 7, 6, 5, 4, and 3 factors. Successive analyses revealed problems in the number of items per factor, cross‐loadings, *λ* < 0.40, and items without factorability for the analysis. Thus, a psychometrically adequate three‐factor solution was reached, confirmed by parallel analysis (which demonstrated that the fourth factor was spurious), by the adequate eigenvalue (> 1), all items with *λ* > 0.40, there were no cross‐loadings, and the total explained variance of the construct was 48.7% (Table 2).

**TABLE 2:** Descriptive data of the factor loadings of the items, eigenvalue, variance explained by factor and total variance explained of the KAP instrument after AFE.

Items	Knowledge	Attitudes	Practices
CBCT21. The presence of metallic restorations may affect the image quality of cone beam computed tomography	**0.85** *	−0.05	0.07
CBCT23. The presence of metal posts may affect the image quality of cone beam computed tomography	**0.93** *	0.04	−0.02
CBCT26. The presence of metallic materials (e.g., posts, restorations) may affect the evaluation of the cone beam computed tomography image	**0.88** *	0.02	−0.04
CBCT2. I consider my knowledge of cone beam computed tomography sufficient for my clinical practice	0.02	**0.82** *	0.00
CBCT3. I have been using cone beam computed tomography for many years	0.12	**0.58** *	0.06
CBCT4. I believe I am capable of interpreting cone beam computed tomography in my clinical practice	−0.04	**0.84** *	−0.00
CBCT7. I believe I am capable of interpreting cone beam computed tomography without a radiologist's report in my clinical practice	−0.01	**0.65** *	0.01
CBCT6. I consider it essential to request cone beam computed tomography in root canal retreatment cases	0.03	−0.06	**0.54** *
CBCT10. I consider it essential to request cone beam computed tomography in cases of dental trauma	−0.08	0.09	**0.52** *
CBCT13. I consider it essential to request cone beam computed tomography in cases of complex root canal anatomy	0.05	0.04	**0.53** *
CBCT19. I consider it essential to request cone beam computed tomography in suspected cases of root fracture	0.07	−0.14	**0.55** *
CBCT31. I request cone beam computed tomography to locate the MB2 canal in upper molars	−0.05	0.15	**0.47** *
CBCT39. I request cone beam computed tomography to evaluate fractured instruments	−0.05	0.09	**0.50** *
Eigenvalue	3.561	2.330	1.784
Variance explained by factor	18.50%	17.30%	12.90%
Total variance explained	48.7%		

* λ > 0.40.

### 3.5 Confirmatory Factor Analysis

**FIGURE 2:**
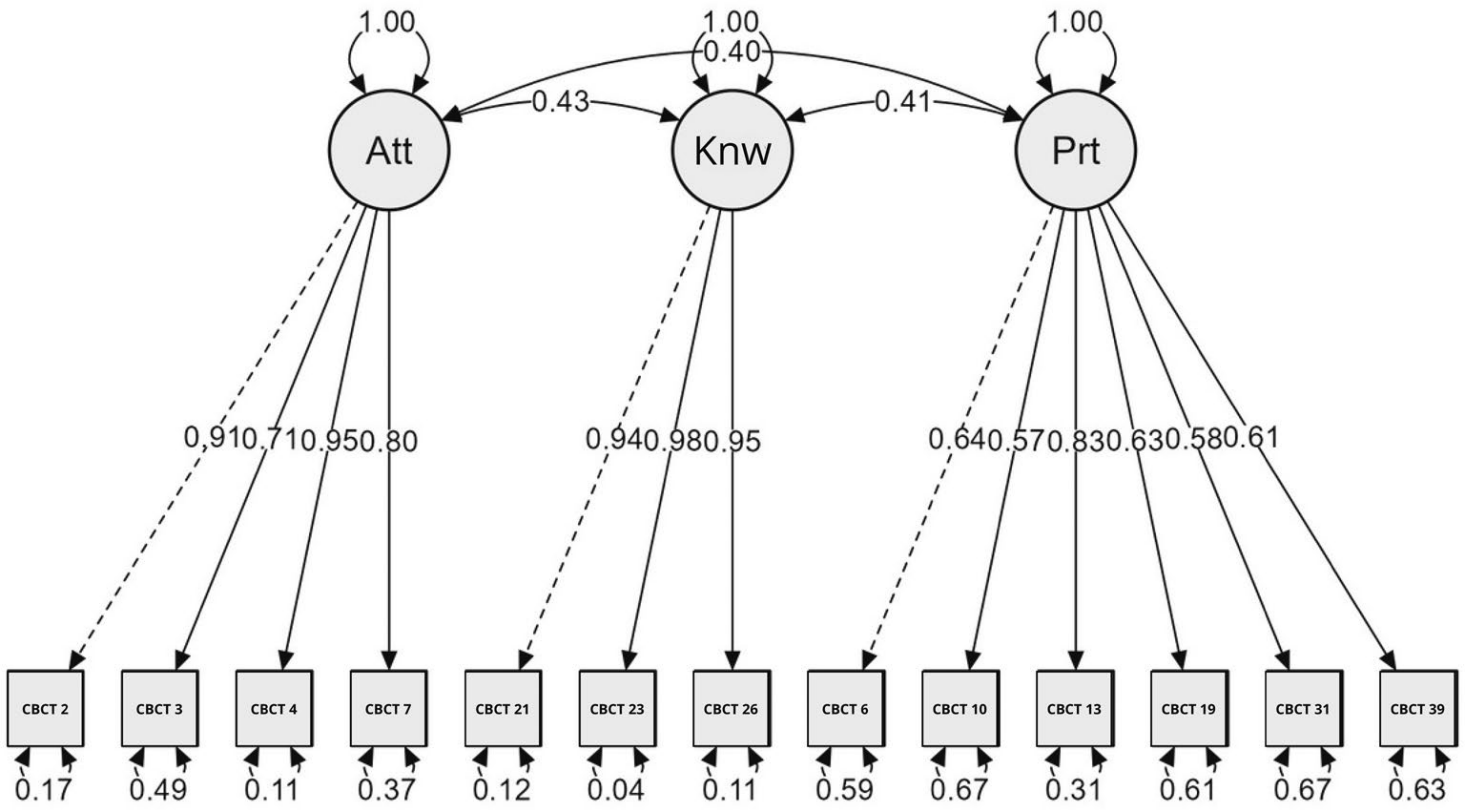
Confirmatory factor analysis diagram of the knowledge, attitudes and practices instrument on cone beam computed tomography. The circles represent the factors. The arrows between the circles represent the correlation between the factors. The values on the arrows between the circle and the square are the factor loadings of the items. The value below the square is the measurement error of the item.

CFA was conducted to confirm the factor structure obtained in the EFA for the instrument. It demonstrated excellent factorial fit (*χ* ^2^ (df) = 1.31; CFI = 0.99; TLI = 0.99; RMSEA = 0.04 [90% CI = 0.00–0.06]; SRMR = 0.05). The factor loadings were satisfactory for all questionnaire items in their respective factors (*λ* > 0.57). The scale factors showed a statistically significant correlation (*p* < 0.001) with each other. Specifically, a positive and moderate correlation was identified between the factors “knowledge” and “attitudes” (*r* = 0.43), “knowledge” and “practices” (*r* = 0.41), and “attitudes” and “practices” (*r* = 0.40) (Figure 2).

### 3.6 Internal Consistency

Reliability analysis was performed using internal consistency, specifically McDonald's omega coefficient (*ω*), considering the samples and factors defined in the CFA. The instrument showed internal consistency adequate for all factors: Knowledge (*ω* = 0.93 [95% CI = 0.91–0.94]), Attitudes (*ω* = 0.86 [95% CI = 0.83–0.89]), and Practices (*ω* = 0.71 [IC 95% = 0.66–0.77]), indicating good reliability of the instrument.

### 3.7 Discriminant Analysis

Discriminant analysis comparing endodontists' scores based on length of experience and level of education was performed using the final structure of the questionnaire, with data from 506 participants. The final CBCT questionnaire consisted of 13 items: Knowledge 3 items (minimum 3 points and maximum 15 points), Attitudes 4 items (minimum of 4 points and maximum 20 points), and Practices 6 items (minimum of 6 points and maximum 30 points) (Appendix S1).

The scores per factor were compared in relation to the length of experience of the endodontists, divided into those with shorter experience in the field (1 year or less to 6 years; *n* = 270) and those with longer experience in the field (7 years or more; *n* = 236). The scores on the “attitudes” factor showed statistical significance (*p* < 0.001) and a moderate effect size (rrb = 0.40) between the two groups, with the group with longer experience as an endodontist (7 years or more) presenting a higher score, that is, the longer experience as an endodontist, the better their attitudes toward CBCT. More information is presented in Table 3.

**TABLE 3:** Descriptive analysis and Mann–Whitney *U* test results comparing knowledge, attitude, and practice scores from the CBCT questionnaire between those who have shorter experience as endodontists (1 year or less to 6 years) and those who have longer experience as endodontists (7 years or more).

CBCT	1–6 years (*n* = 270)	7 years—more (*n* = 236)	Statistic	Effect size (rank‐biserial correlation)
Attitudes	13.77 (3.19)	16.01 (3.49)	*U* = 44641.50***	0.40
Knowledge	13.44 (2.15)	13.62 (1.95)	*U* = 32974.50	0.04
Practices	25.43 (3.21)	25.72 (3.22)	*U* = 33282.50	0.05

*** *p* < 0.001.

In comparisons between the scores on the questionnaire and the endodontist's level of education, the Kruskal–Wallis test revealed statistically significant differences in the fields of attitudes (H (3) = 107.54, *p* < 0.001) and knowledge (H (3) = 14.32, *p* < 0.002); the effect size for the knowledge factor was small (*ε* ^2^[H] = 0.03) and for the “attitudes” factor it was medium (*ε* ^2^[H] = 0.21), which may infer that the level of education explains a small portion of the variance in the knowledge factor scores. The variance in “attitudes” factor scores is moderately explained by level of education. Pairwise comparisons using Dunn's post hoc test indicated statistical significance in the lower scores of specialists on the “attitudes” factor compared to endodontists with master's, doctorate, and postdoctoral degrees. On the “knowledge” factor, the lower scores of specialist endodontists compared to endodontists with master's and doctorate degrees were statistically significant. The complete data analysis is shown in Table 4.

**TABLE 4:** Descriptive analysis and result Kruskal–Wallis test and Dunn's post hoc test comparing the knowledge, attitude and practice scores of the CBCT questionnaire, and the educational levels (specialization, master's, doctorate, postdoctoral) of endodontists.

CBCT	Specialization (*n* = 318)	Master's degree (*n* = 110)	Doctorate (*n* = 71)	Postdoctoral (*n* = 7)	Statistic	Effect size (rank *ε* ^2^)
Attitudes	13.64^a^, ^b^, ^c^ (3.32)	16.44^b^ (2.79)	17.27^a^ (3.00)	17.86^c^ (2.27)	H (3) = 107.54***	0.21
Knowledge	13.31^a^, ^b^ (2.16)	13.82^b^ (1.72)	14.04^a^ (1.97)	13.57 (1.51)	H (3) = 14.32***	0.03
Practices	25.36 (3.22)	25.82 (3.11)	26.09 (3.35)	26.00 (3.16)	H (3) = 4.08	0.01

^a^ Significant difference between specialization and doctorate.

^b^ Significant difference between specialization and master's degree.

^c^ Significant difference between specialization and postdoctoral.

*** *p* < 0.001.

## 4 Discussion

This study aimed to develop and validate evidence of validity and reliability of an instrument for measuring KAP of endodontists regarding CBCT. Previous studies in the literature evaluated many characteristics of CBCT, but the instruments used in these evaluations there was not a proper validation process for the questionnaires applied, compromising the results and the inferences made [3, 4, 5, 6, 7, 10, 11, 12, 13, 14, 15]. Another point observed among the studies and reported in the literature [26], was the lack of conceptual clarity of the points raised, with no definition of the characteristics analysed, a mixture of concepts, such as descriptive analyses providing inferences about characteristics that cannot be directly measured (for example: knowledge and attitudes).

The evaluation of the psychometric properties of questionnaires contributes to the quality of the outcomes evaluated in research, highlighting the role of psychometrics in the development and application of constructs that evaluate characteristics that cannot be observed and measured directly (such as KAP) [8, 9]. The different evaluation methods applied in psychometrics aim to prevent the created instrument from containing irrelevant items or that the items represent poorly the construct [27]. This makes the instruments created or replicated more reliable and valid. Results obtained in studies without due methodological rigor have a serious risk of inconsistent, vague, or biased inferences that can lead to erroneous conclusions [8, 9]. In Dentistry literature there is a lack in the methodology for constructing questionnaires applied in research [3, 4, 5, 6, 7, 10, 11, 12, 13, 14, 15]. On the other hand, some studies validated the questionnaires used, to make more reliable inferences [28, 29].

For constructing the instrument, the proposed items were developed after an extensive review of the literature and information from experts in endodontics, radiology, and psychometrics [19, 27], prioritizing relevant points discussed on CBCT related to the KAP of endodontists and methodological adaptations of the construct. The choice of standardization of responses through the *Likert* scale was a convenient way to measure variables not directly observable, since the intention was to quantify the responses [26, 30]. Content validity, used to evaluate items and determine the need for adjustments or elimination, was conducted by dentists with at least a doctoral degree in endodontics or radiology. Response analysis was interpreted, and the items retained had notable clarity, relevance, and logical relevance to the constructs, as recommended in the literature [19, 27].

Understanding the perception of the target group that will be assessed with the instrument is essential [27]. Therefore, semantic analysis was conducted with endodontists of different ages and educational levels, ensuring that the instrument's items were comprehensible for the intended purpose and intelligible to endodontists with different levels of skills [17]. After this stage, some items were adjusted for better comprehension.

The next step consisted of construct analysis, used to test whether all items resulting from the previous steps actually measured the construct and which items needed to be removed [30]. Once the questionnaire has been created, CFA is performed to determine how well the model fits the data [30]. The indices evaluated in the CFA were adequate for the questionnaire. Internal consistency estimates how closely a set of items is related [30]. Thus, it was estimated for each factor separately and demonstrated good results.

When asked about any prior training in CBCT, the majority (74.11%) of endodontists responded that they had received training during their specialization. These findings are more positive than those found by Cheung et al. [6] in their global survey of endodontists, in which a small portion of the sample had received CBCT training during their undergraduate degree in dentistry or specialization in endodontics. Most endodontists in this study did not have a CBCT scanner at their workplace, which may be attributed to the high cost of the scanner in Brazil. Among other studies, most endodontists had the scanner at their workplace [3, 4, 5, 6, 12, 13, 14, 15].

When comparing scores on the KAP factors and length of service as an endodontist regarding CBCT, this study found better attitudes among endodontists with longer experience (7 years or more), but this did not influence their knowledge and practices. Education level partially explained better attitudes and knowledge among endodontists regarding CBCT, with those with only a specialization presenting worse attitudes and less knowledge, but this did not interfere with practices. Better attitudes toward CBCT according to longer experience may be related to the greater complexity of the cases endodontists receive during consultations, and consequently, a greater need to request more detailed examinations. The fact that higher educational levels influence endodontists' knowledge may be due to greater in‐depth studies of CBCT, also leading to better attitudes, given their greater knowledge.

This study focused on evaluating the psychometric properties of the constructed questionnaire and making inferences regarding the length of experience and level of education of endodontists in relation to CBCT. The analysis demonstrated that the created construct is valid and reliable for measuring the proposed measures. One limitation is the lack of focus groups and interviews with experts during the item development stage [27]. No studies in the literature have conducted similar research and psychometric analysis. We believe that our work can contribute to a better assessment of endodontists' knowledge, attitudes, and practices regarding CBCT, as the questionnaire created can be used to develop new research.

Further analyses must be conducted to utilize the questionnaire and establish reliable measures. For studies in Brazil, the instrument should be tested in different regions, given the country's continentality. Cross‐cultural adaptation of the construct is necessary for use in other countries/languages. In both cases, validity and reliability checks, as well as measurement invariance, are required to establish comparisons between countries and other samples. Future studies may establish comparisons with the results found and explore other demographic features, as well as discuss the relationship between the use of CBCT and the influence of socioeconomic conditions. Once again, to this end, further studies should examine measurement invariance across relevant variables before conducting comparisons between groups.

## 5 Conclusion

The knowledge, attitudes and practices instrument developed followed all the necessary steps for validating measurement instruments, demonstrating good evidence of content, discriminant and construct validity, as well as adequate internal consistency.

Longer experience as an endodontist resulted in more positive attitudes toward CBCT, and the level of education influenced these attitudes and knowledge.

## Author Contributions

**Lisa Morais Fernandes Oliveira:** conceptualization, investigation, formal analysis, methodology, resources, validation, visualization, writing – original draft preparation, writing – review and editing. **Francielle Silvestre Verner:** conceptualization, investigation, methodology, resources, visualization, writing – review and editing. **Pedro Henrique Berbert de Carvalho:** conceptualization, data curation, formal analysis, investigation, methodology, resources, supervision, validation, visualization, writing – review and editing. **Rafael Binato Junqueira:** conceptualization, investigation, methodology, project administration, resources, supervision, visualization, writing – review and editing.

## Conflicts of Interest

The authors declare no conflicts of interest.

## Supporting information


**Appendix S1:** Final CBCT questionnaire consisting of 13 items: Knowledge 3 items, Attitudes 4 items, and Practices 6 items.

## Data Availability

The data that support the findings of this study are available from the corresponding author upon reasonable request.
