# Supplementary material for: Development and Validation of an Instrument to Assess Endodontists' Knowledge, Attitudes, and Practices Regarding Cone‐Beam Computed Tomography
Source: Aust Endod J. 2026 Mar 29;52(2):498–506. doi: 10.1111/aej.70075 (PMC13436305; doi:10.1111/aej.70075)
Supplement: Supplementary file 1 [file AEJ-52-498-s001.docx]

**CONE BEAM COMPUTED TOMOGRAPHY**

**The sentences were translated literally into English. Therefore, cross-cultural adaptation is necessary for the questionnaire to be used effectively. The original questionnaire in Portuguese can be requested from the authors for cross-cultural adaptation.**

- **Knowledge**

**1. The presence of metallic restorations may affect the image quality of cone beam computed tomography.**

I strongly agree

I agree

I neither agree nor disagree

I disagree

I strongly disagree

**2. The presence of metal posts may affect the image quality of cone beam computed tomography.**

I strongly agree

I agree

I neither agree nor disagree

I disagree

I strongly disagree

**3. The presence of metallic materials (e.g., posts, restorations) may affect the evaluation of the cone beam computed tomography image.**

I strongly agree

I agree

I neither agree nor disagree

I disagree

I strongly disagree

- **Attitudes**

**4. I consider my knowledge of cone beam computed tomography sufficient for my clinical practice.**

I strongly agree

I agree

I neither agree nor disagree

I disagree

I strongly disagree

**5. I have been using cone beam computed tomography for many years.**

I strongly agree

I agree

I neither agree nor disagree

I disagree

I strongly disagree

**6. I believe I am capable of interpreting cone beam computed tomography in my clinical practice.**

I strongly agree

I agree

I neither agree nor disagree

I disagree

I strongly disagree

**7. I believe I am capable of interpreting cone beam computed tomography without a radiologist's report in my clinical practice.**

I strongly agree

I agree

I neither agree nor disagree

I disagree

I strongly disagree

- **Practices**

**8.** **I consider it essential to request cone beam computed tomography in root canal retreatment cases.**

I strongly agree

I agree

I neither agree nor disagree

I disagree

I strongly disagree

**9. I consider it essential to request cone beam computed tomography in cases of dental trauma.**

I strongly agree

I agree

I neither agree nor disagree

I disagree

I strongly disagree

**10. I consider it essential to request cone beam computed tomography in cases of complex root canal anatomy.**

I strongly agree

I agree

I neither agree nor disagree

I disagree

I strongly disagree

**11. I consider it essential to request cone beam computed tomography in suspected cases of root fracture.**

I strongly agree

I agree

I neither agree nor disagree

I disagree

I strongly disagree

**12. I request cone beam computed tomography to locate the MB2 canal in upper molars.**

I strongly agree

I agree

I neither agree nor disagree

I disagree

I strongly disagree

**13. I request cone beam computed tomography to evaluate fractured instruments.**

I strongly agree

I agree

I neither agree nor disagree

I disagree

I strongly disagree
